# Immunomodulatory effects of β-defensin 2 on macrophages induced immuno-upregulation and their antitumor function in breast cancer

**DOI:** 10.1186/s12865-022-00527-y

**Published:** 2022-11-02

**Authors:** Sonam Agarwal, Anita Chauhan, Khushwant Singh, Kunal Kumar, Rupinder Kaur, Marilyn Masih, Pramod Kumar Gautam

**Affiliations:** Department of Biochemistry, All India Institute of Medical Sciences, New Delhi, 110029 India

**Keywords:** Macrophages, β-defensin 2, Cytokine profiling, Anti-tumor function, Oxidative stress

## Abstract

**Background:**

Macrophages are mononuclear CD34^+^ antigen-presenting cells of defense mechanism and play dual roles in tumor burden. The immunomodulatory and their antitumor function of β-defensin 2 is still unclear, despite the accumulating evidence of the response in infection. So, the aim of present study is to elucidate the role of β-defensin 2 on the level of ROS, cytokines, chemokine expression in macrophages and antitumor function in breast cancer.

**Method:**

Swiss albino mice were used to harvest PEC macrophages and C127i breast cancer cells line for tumor model was used in this study. Macrophages were harvested and characterized by flow-cytometry using F4/80 and CD11c antibodies. MTT was performed to estimate cytotoxicity and dose optimization of β-defensin 2. Oxidative stress was analyzed by H_2_O_2_ and NO estimation followed by iNOS quantified by q-PCR. Cytokines and chemokines estimation was done using q-PCR. Co-culture experiment was performed to study anti-tumor function using PI for cell cycle, Annexin –V and CFSE analysis for cell proliferation.

**Results:**

PEC harvested macrophages were characterized by flow-cytometry using F4/80 and CD11c antibodies with the purity of 8% pure population of macrophages. It was found that 99% of cells viable at the maximum dose of 100 ng/ml of β-defensin 2 in MTT. Levels of NO and H_2_O_2_ were found to be decreased in β-defensin 2 as compared to control. Expression of cytokines of IFN-γ, IL-1α, TNF-α, TGF-βwas found to be increased while IL-3 was decreased in β-defensin 2 group as compared to control. Levels of chemokines CXCL-1, CXCL-5 and CCL5 increased in treated macrophages while CCL24 and CXCL-15 expression decreased. Adhesion receptor (CD32) and fusion receptor (CD204) were decreased in the β-defensin 2 group as compared to control. Anti-tumor experiment was performed using co-culture experiment apoptosis (Annexin-V) was induced, cell cycle arrest in phage and cell proliferation of C127i cells was decreased.

**Conclusion:**

This is the first report of β-defensin 2 modulates macrophage immunomodulatory and their antitumor function in breast cancer. β-defensin 2 as a new therapeutic target for immunotherapy as an adjuvant in vaccines.

## Background

Macrophages are mononuclear CD34^+^ phagocytic cell known as potent antigen presenting cells of defense mechanisms which can promote specific immunity by recruitment and activation of different immune cell towards infection and malignancies [1]. Macrophages are classified according to their phenotype and cytokine release such as M1, M2, M2a, M2b, M2c, M2d and tumor associated (TAMs) [2]. Upon phagocytosis, it displays antigen via MHC class I and II bound antigen to T lymphocytes for effective response towards infection and cancer cell specific cell-mediated immunity. In different infective milieu and upon antigen encounter and anchoring with the cell via receptors, releases of a vast range of cytokines include tumor necrosis factor (TNF), IL-1β, IL-6, IL-8, and IL-12. Additionally, the contact of the T cell receptor with an antigen-bound MHC is necessary for the first signal (recognition), and the CD80 or CD86 on the antigen-presenting cell, as well as CD28 and CTLA-4 on the responding T cell, supply the second signal. [3]. It has been demonstrated that the interaction between B7 and CD28 produces a crucial signal for T cell activation.

Immunologically, defensins are associated with the disturbance of the numbers and function of peri-and intrafollicular inflammatory cells, and this disturbance is more abundant during the active stage of the disease. Defensins are a family of small 18–55 amino acids residues, cysteine-rich, cationic proteins expressed predominantly in innate immune cells such as neutrophils and epithelial cells [4]. Their vast research has confirmed their significance in innate immunity as a key host-protective factor against bacterial, viral, and fungal infections, as well as their broad antibacterial properties and diverse immunomodulatory actions. [5]. β-defensin regulates the chemotactic activity for numerous innate immune cells, which also stimulate the release of secrete cytokines from other cells [6]. However, they can also capable of reduce inflammation by specifically attaching to molecular patterns associated with microbes. These patterns have ability to stimulate the expression of β-defensins in gingival epithelial cells, while many bacterial species are found to differ significantly from one another. Simultaneously these finding suggest that a complex paradigm of a host-defense related function in maintenance of bacterial homeostasis and pathogen response [7].

β-defensin 2 are plausible molecules for stimulating not only innate immune but also adaptive immune responses [8]. It activates the immune system by modulation of signaling pathways and inflammatory response [9]. Recent studies have demonstrated that β-defensin 2 can modulate immune responses in various diseases [10]. Apart from their antimicrobial activities, β-defensin 2 is also involved in a variety of other activities, including pathogens killing, cell activation, proliferation, regulation of cytokine/chemokine production, migration, differentiation, angiogenesis, and wound healing. Moreover, they also participate in tumorigenesis, due to their proliferative or suppressive properties relative to different cells [9]. Mei et al. reported that β-defensin 2 may act as a positive regulator, promoting anti-tumor responses of T and NK cell in vivo, therefore it may be useful in immunotherapy [11]. NK cells and CD8^+^ T cells attack against malignancies, while other cells accumulate into the tumor. This imbalance in the inflammatory cells, leading to a collapse of the immune surveillance and inflammatory cells, is not only restricted to the site but promotes tumor. It is also noticed to occur in the spleen, lymph nodes, and the peripheral blood cells affected in tumor patients. A gap in the knowledge of immunomodulatory and tumoricidal function of macrophages with a crosstalk with β-defensin 2.

However, few reports of β-defensin 2 in immunomodulation have been extensively studied and their involvement in other processes such as antitumor activity against cancer remaining unknown. So, the aim of this study was to evaluate the immunomodulatory function of β-defensin 2 on macrophages and to elaborate the antitumor activity on breast cancer. We analyzed the impact of β-defensin 2 on the level of oxidative stress, expression of cytokines and chemokines gene of macrophages. Consequently, the antitumor effect of β-defensin 2 on breast cancer during the active state of the macrophages was also determined in the current study.

## Results

### Isolation and characterization of macrophages (Fig. 1)

**Fig. 1:**
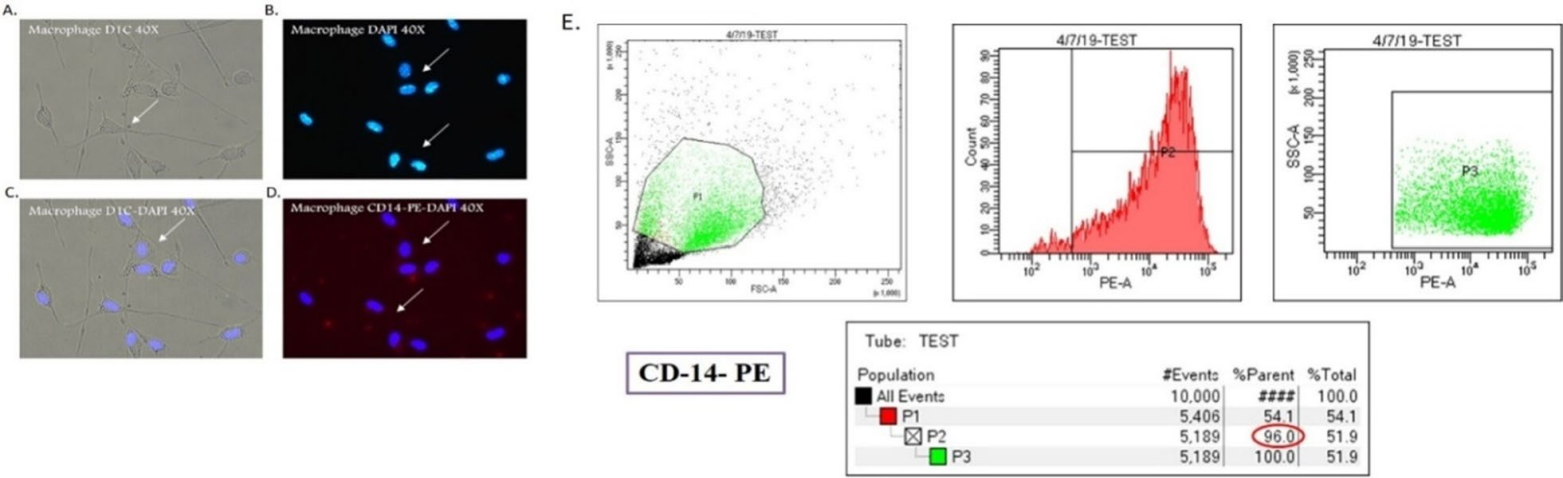
Characterization of macrophages isolated from mice peritoneal cavity based on CD-14^+^ expression through Flow cytometry(E). The cells were gated based on CD-14^+^ expression. Percentage refers to the mean of cell subpopulations gated on the total population of macrophage cells. Representative FACS means fluorescence intensity (MFI) histograms and mean MFI ± SEM of CD14

Normal macrophages were harvested from Swiss albino mice as peritoneal exudates cell by peritoneal lavage with chilled PBS and incubated for 2 h in plastic petri dishes (Tarson, India). After 2 h, adherent cells were collected and stored in media, washed thrice in serum free media at 1500 rpm for 10 min by centrifugation. Flow cytometry was performed for quantification and characterization of harvested cells (Fig. 1E**)**. It was found that more than 96% cells ofF4/80, CD11c positive are labeled with PE and further utilized for ROS estimation and gene expression analysis.

### Immunocytochemistry (ICC) (Fig. 2)

**Fig. 2:**
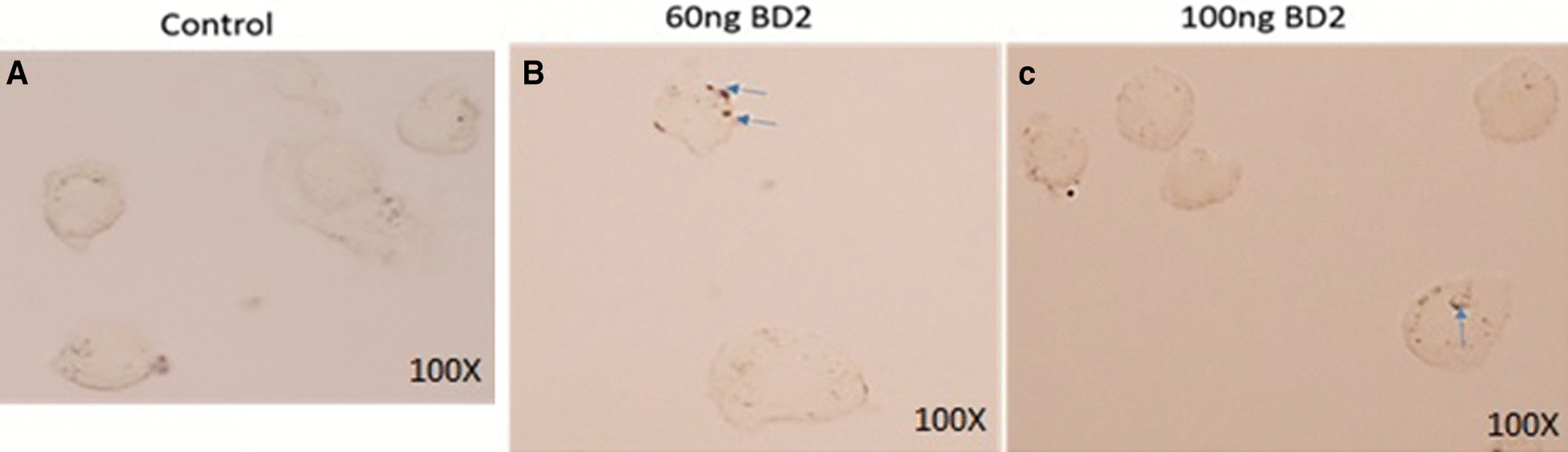
Localization of BD 2 in mice isolated peritoneal macrophage cells after 24 h of treatment

ICC was done to localize the CD14 receptor, which is a marker of macrophages. The β defensins-2 (BD-2) internalization/ phagocytosis by macrophages ICC assay were done. Purified macrophage cells were incubated with a 100 ng/ml dose of BD-2 for 24 h. Slides were prepared as per protocol followed by monoclonal antibodies. Figure 2 shows that internalization of BD-2 in macrophages uniform punctate patch in the cytoplasmic extensions of the cells, while untreated cells there are no any punctate pattern found in the cytoplasmic extension of the cells.

### MTT assay (Fig. 3)

**Fig. 3:**
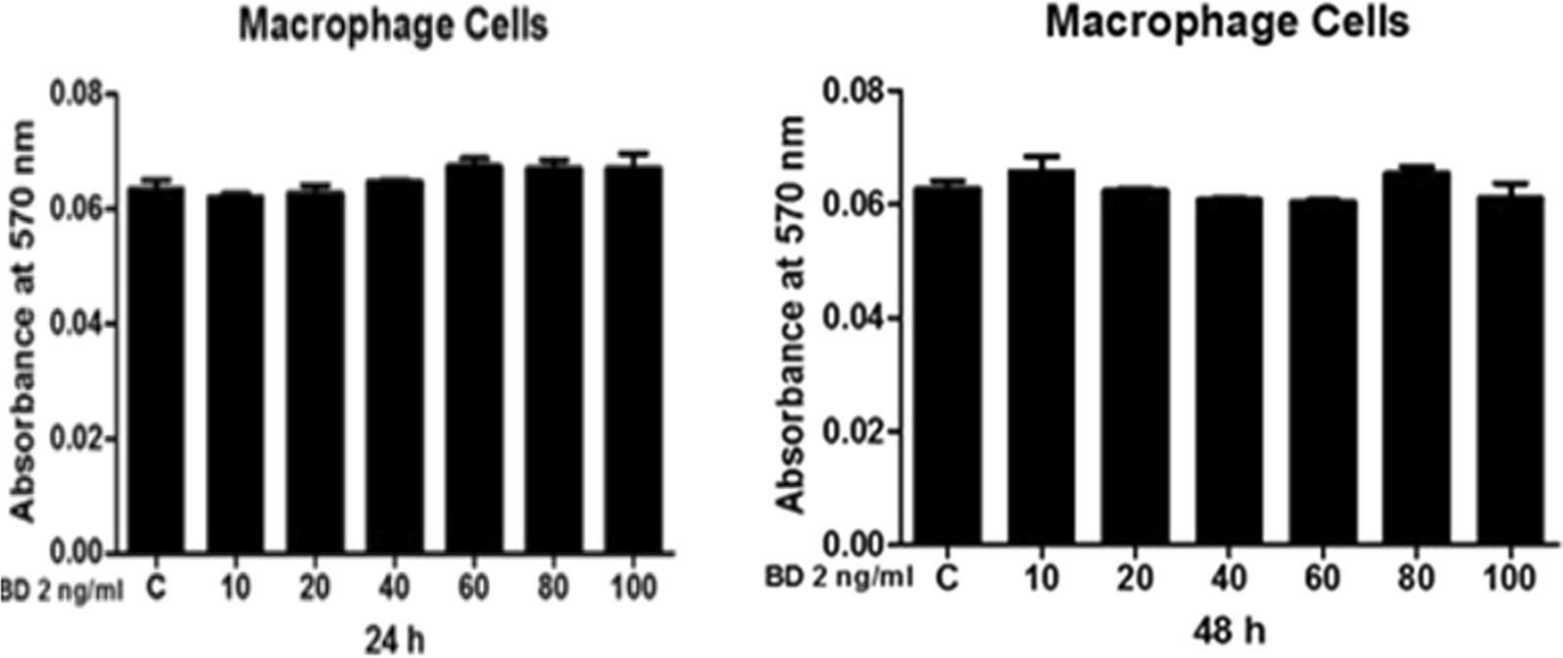
Effects of BD 2 on cell viability of mice isolated peritoneal macrophage cells after 24 and 48 h of treatment. Results are expressed as mean ± SE

Figure 3 shows that the effect of BD 2 on the viability of macrophages, cells were treated with 10–100 ng/ml BD-2 for 24 and 48 h and subjected to MTT assay. Figure 3 shows that no change was found on cell viability of macrophages when treated with BD-2 at various concentrations after 24 and 48 h.

### ROS production on BD-2 treated macrophages (Fig. 4)

**Fig. 4:**
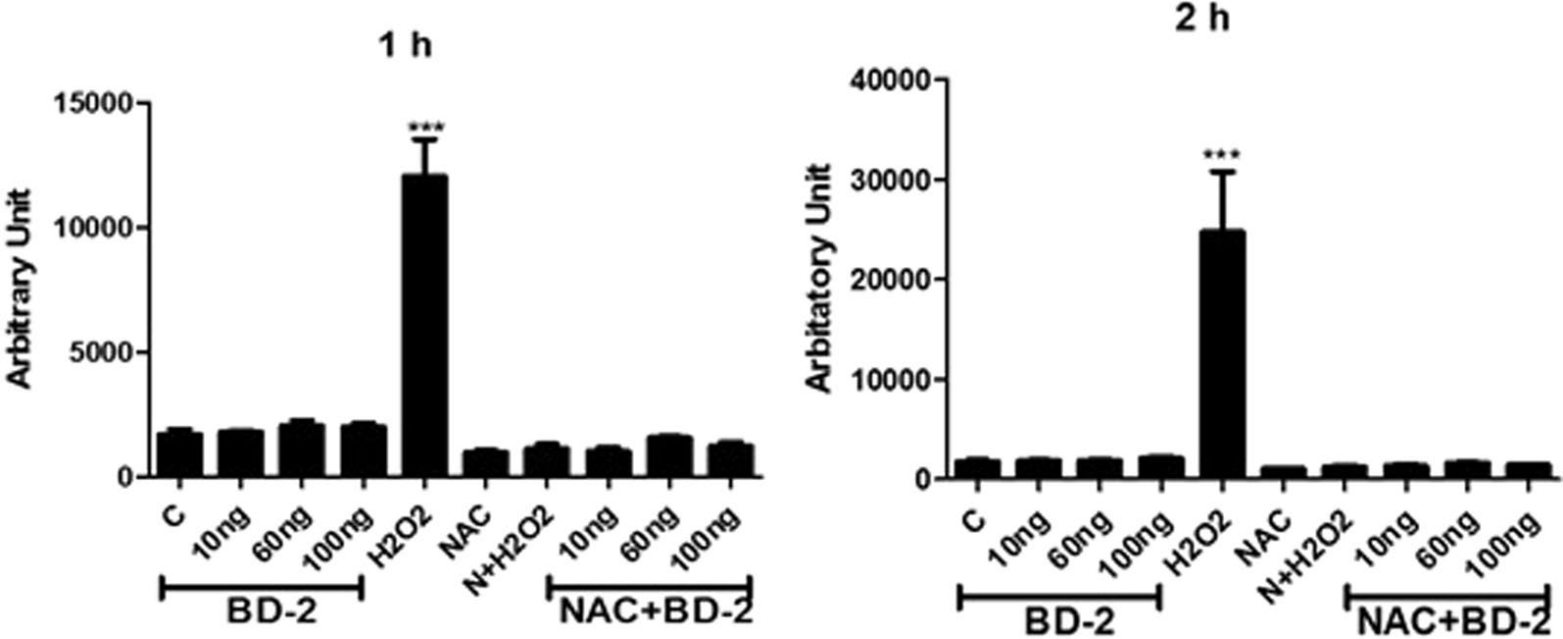
Effects of BD 2 on ROS induction in mice isolated peritoneal macrophage cells after 1 and 2 h of treatment.Results are expressed as mean ± SE. BD-2 (100 ng). Statistical analysis: ***P < 0.001

DCFHDA is a membrane-permeable dye that enters into the cells and is converted into DCF, a fluorescent form when oxidized by ROS present in the cells. The intensity of fluorescence is proportional to the level of ROS present in the cells. Result shows that normally macrophages release ROS to remove the foreign particles present in their surroundings. Therefore, we determined the level of ROS generation with the exposure of BD 2 by DCFHDA assay. Our results revealed that BD 2 could not induce the macrophage to generate ROS at given concentrations in the indicated time points as shown in Fig. 4A–D. Cells were treated with 100 ng/ml of BD-2 for 24 h. Results show that treatment of BD-2 does not induce the release of expression of ROS on normal phenotype of macrophages as compared to LPS treated and medium-only while a positive stimulator H_2_O_2_ stimulates the ROS releases at a significant level. NAC used a negative control for the release of ROS which shows decreased expression of ROS as compared to medium only (Control).

### Nitric oxide measurement (Fig. 5A–C)

**Fig. 5:**
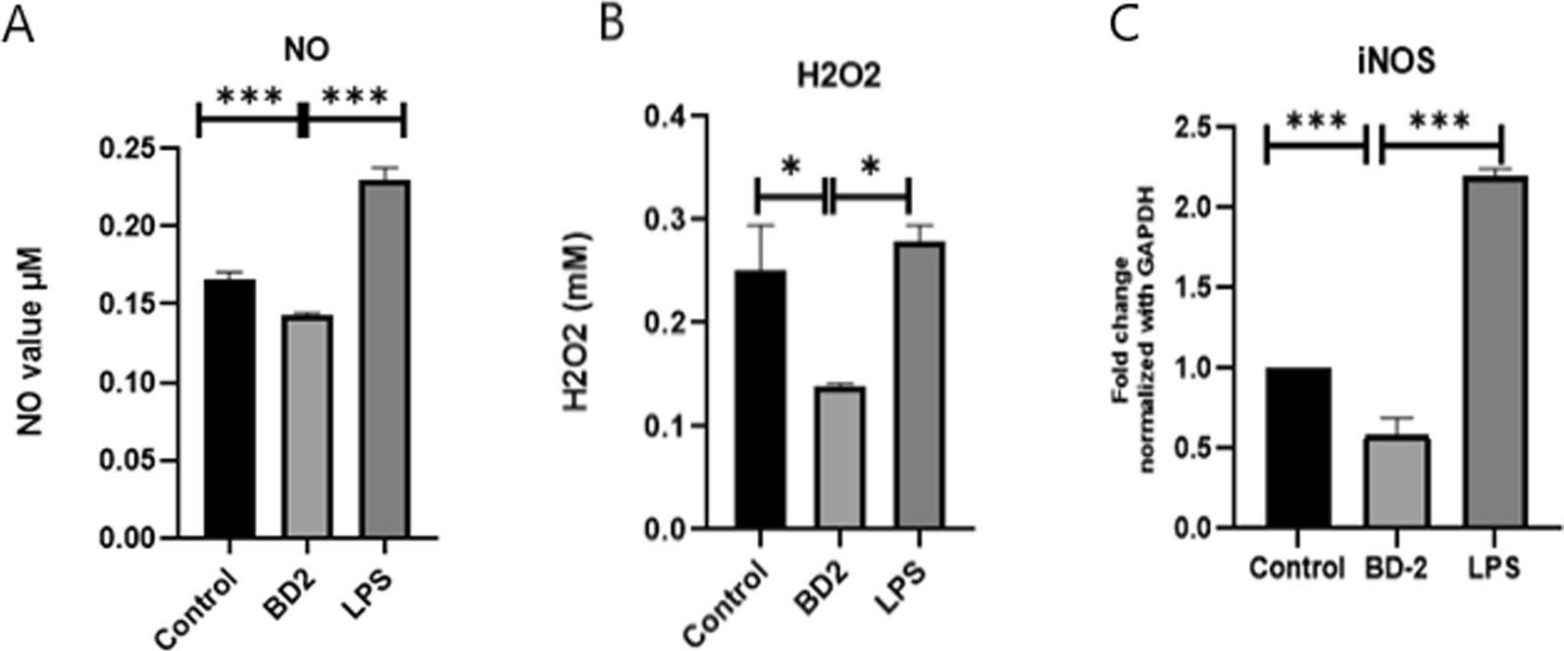
**A**–**C** Effects of BD 2 on the level of H_2_O_2_, NO and iNOS in mice isolated peritoneal macrophage cells after 24 h treatment.Results are expressed as mean ± SE. Statistical analysis: *P < 0.05, ***P < 0.001

The Reactive oxygen species contains NO^−^ and H_2_O_2_ which was estimated by Griess method and TISO_4_ method. The amount of NO production in the supernatant of macrophages after 24 h treatment with β-defensin 2 and LPS was determined with the Griess reagent. During this period, the NO level decreased remarkably in the β-defensin 2 group as compared to the control group (Fig. 5A). It was observed that in other experiments were conducted to examining the effect of β-defensin 2on the production of H_2_O_2_^−^. Production of H_2_O_2_was found to be decreased in β-defensin 2treated group as compared to control (Fig. 5B). mRNA level of iNOS was downregulated in β-defensin 2 treated group as compared to control group (Fig. 5C).

### RT- PCR for selected gene expression on macrophages (Figs. 6 and 7)

**Fig. 6:**
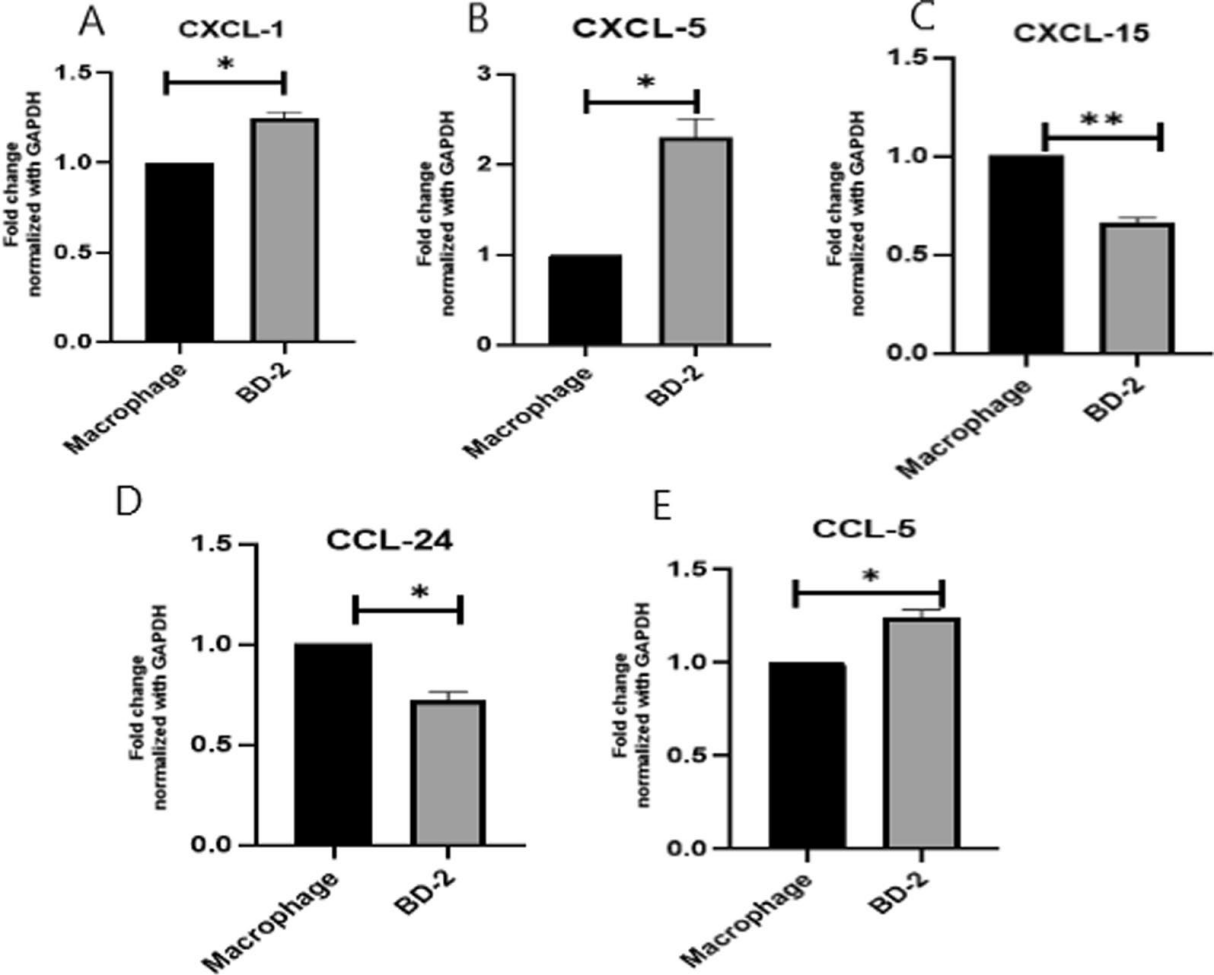
**A**–**E** Effects of BD 2 on the expression of chemokinesin mice isolated peritoneal macrophage cells after 24 h treatment.Results are expressed as mean ± SE. Statistical analysis: *P < 0.05, **P < 0.01

The effect of β-defensin 2 was studied on cytokine andchemokine genes in M1 macrophage with the help of real time PCR. Cytokine and chemokine expression profiles were examined. Macrophages (1 × 10^6^) were purified and characterized in 12 well plates with complete media in triplicate. Cells were treated with 100 ng/ml of BD-2 for 24 h. We have found that BD-2 treated macrophages significantly increased the expression of cytokines IFN-γ, IL-1α, IL-6, TNF-α and TGF-βwhile diminished the level of IL-3, CD32 and CD204as compared to control group (7A and B). In chemokines, we have found increased the level of CXCL-1, CXCL-5 and CCL-5while decreased the level of CXCL-15 and CCL-24as compared to control mice (Fig. 6A–E).

**Fig. 7:**
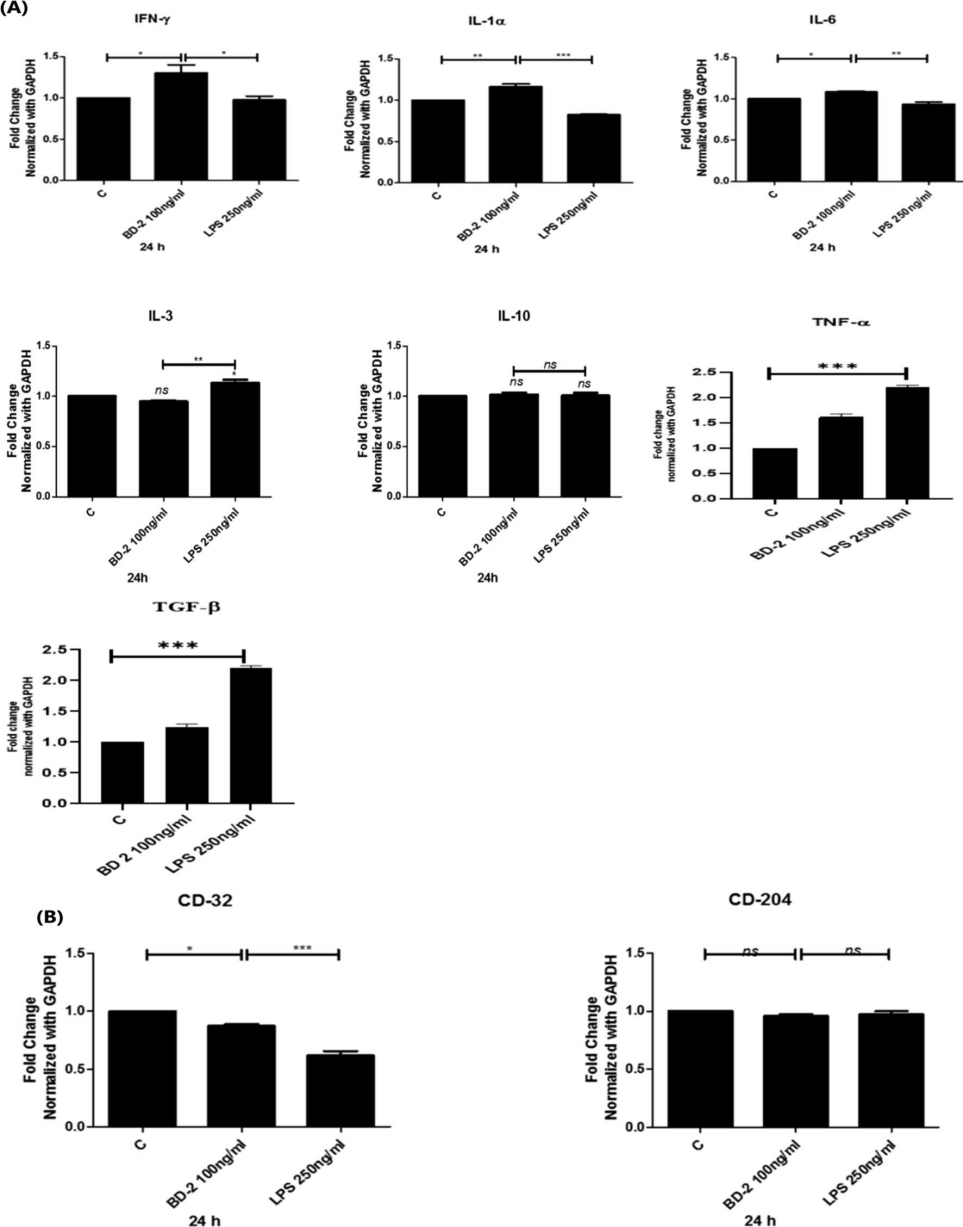
**A**,** B** Effects of BD 2 on the expression of cytokines in mice isolated peritoneal macrophage cells after 24 h treatment. Results are expressed as mean ± SE. *P < 0.05, ***P < 0.001, **P < 0.01

### Apoptotic assay (Fig. 8)

In comparison to macrophage co-cultured and unstimulated c127i, co-culture of c127i with macrophage decreased apoptosis in c127i cells from 5.37 to 4.76%, and β-defensin 2 activated macrophages further decreased apoptosis to 2.66% from 4.76 to 5.37%, respectively. In contrast to unstimulated c127i, there was an increase in late apoptotic cells percent 7.26% from 1.42% in macrophage co-cultured c127i. Macrophage co-cultured c127i when compared to β-defensin 2 activated macrophage co-cultured c127i and to unstimulated c127i, increased percentage of late apoptotic cells 7.26% was observed. At the very same time in c127i cells co-cultured with β-defensin 2, we noticed an increase in the number of dead cells. The dead cell population was enhanced by β-defensin 2 activated macrophages to 10%, up from 5.4% in c127i co-cultured with macrophages and 2.8% in untreated cells. Macrophages increase late apoptotic cell counts but not direct apoptosis in c127i cells, according to the apoptosis analyses. Furthermore, β-defensin 2 stimulated macrophages triggers necrosis or cell death in late apoptotic c127i cells.

**Fig. 8:**
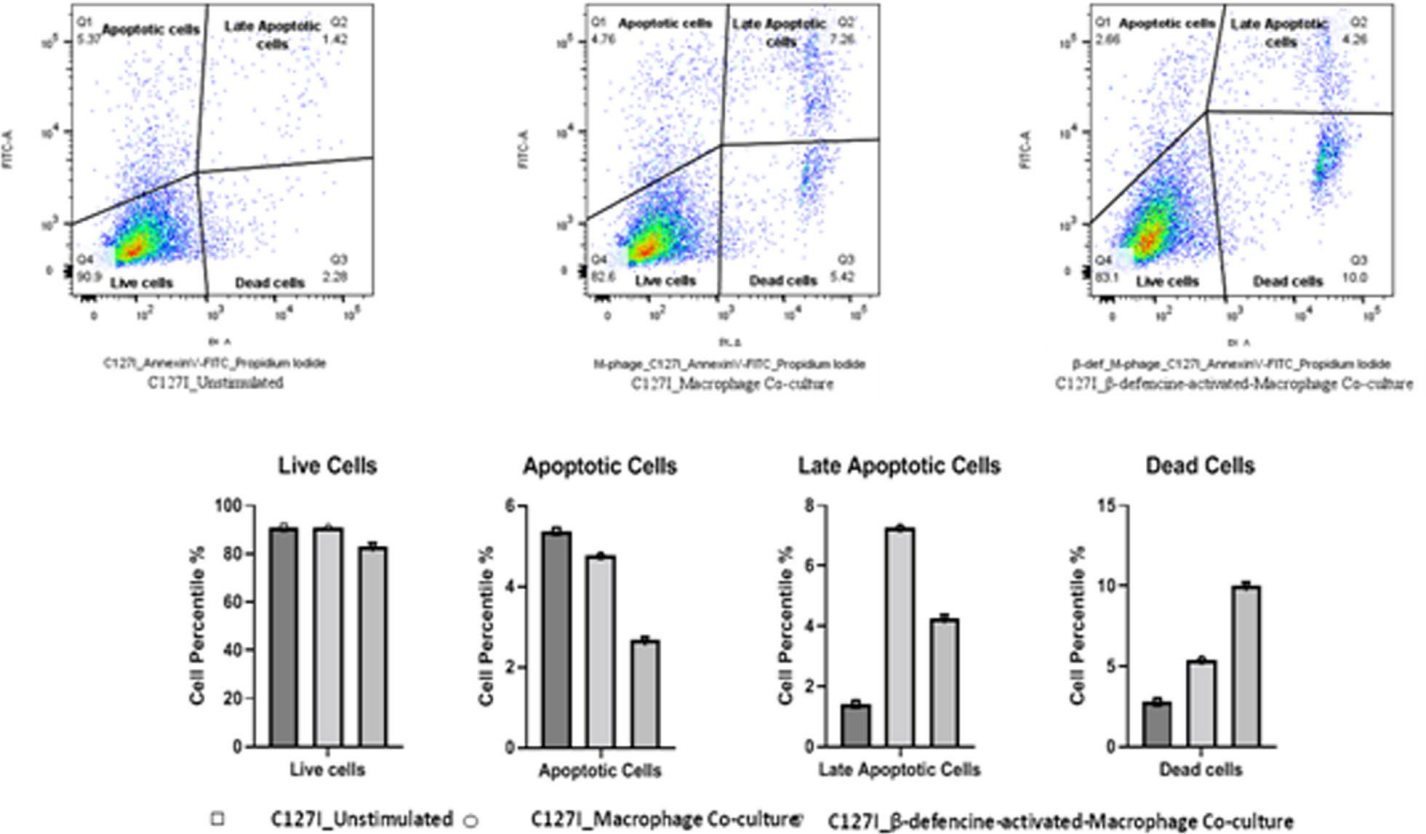
Effects of BD 2 on the apoptotic assay in mice isolated peritoneal macrophage cells after 24 h treatment. Apoptotic and necrotic cells were quantified by flow cytometry, and the different subpopulations were defined as Q1, Annexin V−/PI+, apoptotic cells; Q2, Annexin V+/PI+. Late apoptotic cells; Q3, Annexin V−/PI−, dead cells; and Q4, Annexin V+/PI−, live cells.

### Cell division analysis (Fig. 9A-C)

The number of undivided cells in β-defensin-activated-macrophage co-culture c127i cells was 512, compared to 5906 and 1367 cells in unstimulated and macrophage co-culture c127i cells, respectively. In comparison to the unstimulated and macrophage co-culture c127i cells, β-defensin-activated-macrophage co-culture c127i cells undergo more divisions and cells of the 4th and 5th generations were missing. Furthermore, it was discovered that β-defensin-activated-macrophage co-culture c127i cells had a higher percentage of dead cells (50%) than unstimulated (1.9%) and macrophage co-culture c127i cells (4.4%), respectively. The present findings of CFSE cell proliferation and apoptosis indicate that β-defensin-activated-macrophage cause cell death in c127i cells via necrosis or other alternative mechanism other than direct apoptosis.

**Fig. 9:**
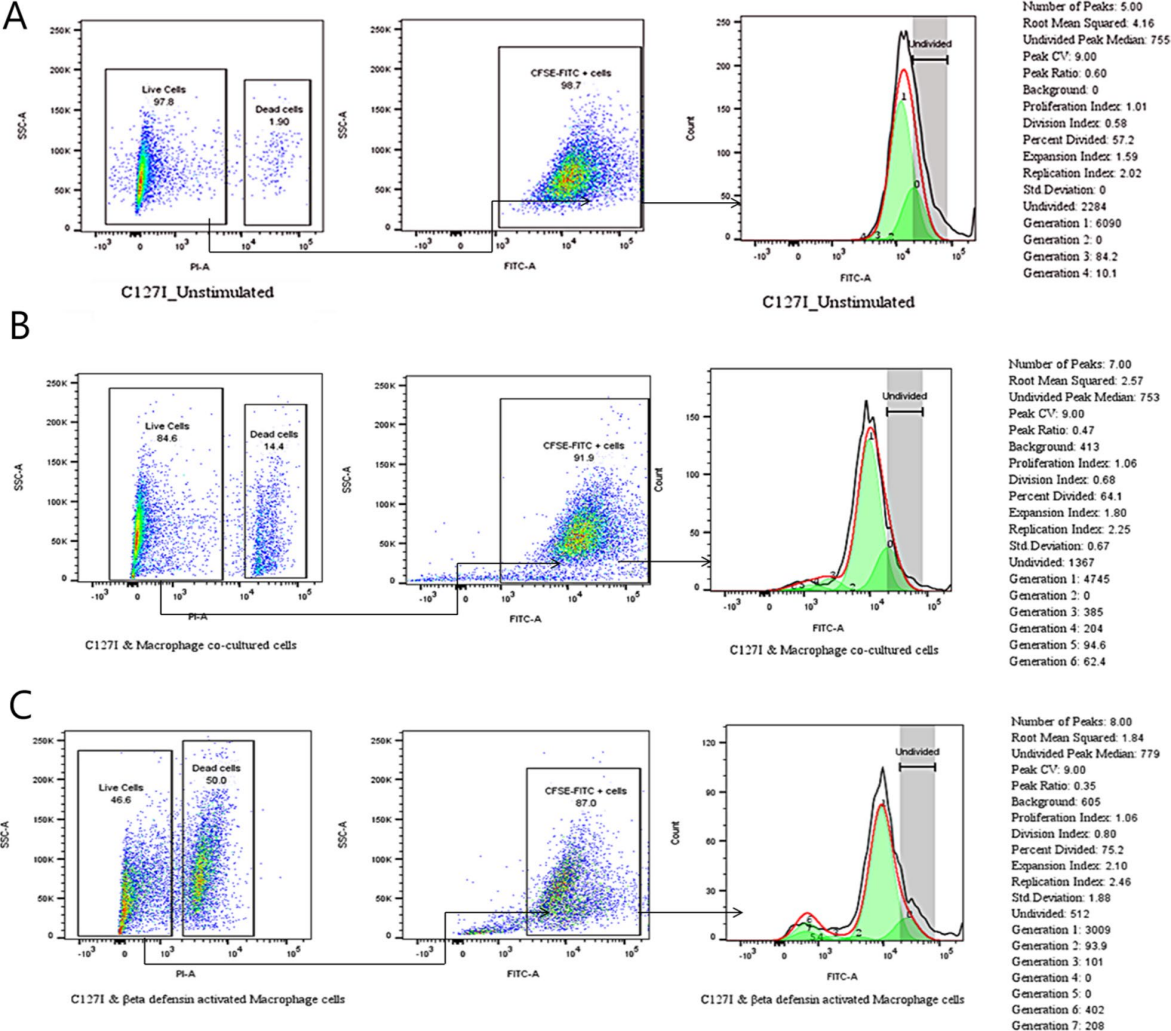
**A**–**C** Effects of BD 2 on the cell division analysis in mice isolated peritoneal macrophage cells after 24 h treatment. Percentages of cells distribution in different generation of the cells were calculated

## Discussion

The immune system is an interactive network of immune cells, lymphoid organs, humoral factors, fibroblasts, tumor vasculature and extracellular matrix make up the stromal environment. In many ways, it is like an army equipped with different weapons to provide protection against infection and malignancies. It has been found that systemic regulation of immune responses is highly impaired when infection greatly increases. During tumor progression, infected cells release various immunosuppressive molecules that suppress immune response directly or activate the host suppressor mechanism in many ways. Tumor actively recruits cells into the tumor microenvironment, including bone marrow-derived mesenchymal stem cells (MSCs), macrophages, NK cell, T cells and GD cells whose function is suppressed in tumor milieu and promotes tumor growth [12, 13]. Several defense proteins expressed by cells and tissue play a cytoprotective role and control infections by recruiting immune cells for pathogen clearance. β-defensins are a family of epithelial cell derived antimicrobial peptides (AMPs) that protect mucosal membranes from microbial challenges [14]. In addition to their antimicrobial activities, they possess other functions; e.g., cell activation, proliferation, regulation of cytokine/chemokine production, migration, differentiation, angiogenesis, and wound healing processes. It has also become apparent that defending levels change with the development of neoplasia. However, inconsistent observations published by various laboratories make it difficult to reach a consensus as to the direction of the dysregulation and role the hBDs may play in various cancers.

We discovered that consensus-driven findings indicate β-defensin 2 not only actively participates in infection, it activates macrophages to perform immunomodulatory and anti-tumor functions too. The effect of β-defensin 2 on macrophages at the dose at maximum 1 ng/ml shows 99% cell viability. It shows that cytoprotective molecules expressed by tissue and immune cell are non –toxic for self cells. Several other published reports support this hypothesis that hsps increases immunomodulatory function and increasing anti-tumor function of tumor associated macrophages by enhancing ROS, cell surface receptors expression such as B7 family receptors, CD54, Cd47, SIRP a, CTLA-4 etc. It also increases the antitumor by increasing il-2, il-4, il-6, tnf-alpha. In some amount ROS production facilities signaling pathway such as NF-kappa b which actively involved in cytokine production. Some data shows that during tumor progression ROS release an increase many fold which facilitates tumor growth.

The most commonly encountered free radicals in cell are hydroxyl radical, superoxide radical, nitric oxide radical, and lipid peroxyl radical. NO and H_2_O_2_ are produced in high quantities from macrophages activated by pro-inflammatory cytokines which leads to cell lesion [15]. NO is synthesized by iNOS, involved in suppression of tumor growth [16]. In accordance with these reports, in the current study, NO and H_2_O_2_, which is expressed by macrophages was found to be reduced when treated with β-defensin 2 as compared to control (Fig. 5), indicating the cytoprotective effects of BD-2.

TNF-*α* is a pro-inflammatory cytokine, plays an essential role in inflammation, apoptosis, lipid metabolism and immune system development [17]. TGF-β is an anti-inflammatory cytokine, higher levels of anti inflammatory cytokine released by activated macrophages which promote tumor suppression leads to the antitumor function. Our results revealed that enhanced the expression of pro-inflammatory cytokines IFN-γ, IL-1α, IL-6, TNF-α, and TGF-β while reduced the expression of IL-3 and IL-10 in BD-2 treated group as compared to control (Fig. 7). A high level of TGF-β has been associated with the regression of growth and survival of tumor which leads to the stimulate apoptosis.

There are several studies reported that higher level of CCL-5 was found in ischemia–reperfusion injuries of brain, lung, heart, kidney, intestine, and skeletal muscle [18–23]. We have shown here that the level of chemokines CXCL-1, CXCL-5 and CCL5 was found higher while CCL24 and CXCL-15 decreased in BD-2 treated group as compared to control (Fig. 6). Higher expression of chemokines was correlated with inflammatory response which leads to the tumor suppression suggesting that BD-2 may act as antitumor function.

## Concluding remarks

Our present study provides the first evidence that BD-2 plays essential role as an antitumor function. M1 macrophages secrete pro-inflammatory cytokines (including TNF-α, IL-6, TGF-β, and IFN-γ) and chemokines to induce apoptosis and reduce cell proliferation which leads to the antitumor function. The main findings of the present study are that BD-2 abridged the level of ROS and NO which is associated with tumor regression. In future research, we hope that we can study the mechanism and learn more about the immunomoutory function of BD-2.

## Method

### Reagents

RPMI-1640 media and FBS were purchased from Gibco, Waltham, Massachusetts, USA. Antibiotics, LPS, DCFHDA, TISO_4_ and H_2_O_2_ were procured from Sigma-Aldrich, St. Louis, Missouri, USA. MTT, DAPI and DABCO were purchased from SRL, India. Anti-mouse CD14-PE (Thermo-Scientific, Waltham, Massachusetts, USA), β-defensin 2 (Prospec, Hamada, Israel) and RNAiso plus (Takara, Japan) were purchased. cDNA synthesis kit and SYBR green were obtained from BIO-RAD, USA. AnnexinV-FITC/PI and CFSE were purchased from Biolegend, San Diego, USA. Anti-mouse CD14 conjugated with PE was purchased from Thermofisher, USA.

### Animals and ethical clearance

Inbred populations of Swiss albino strain of mice of either sex and at 8–12 weeks of age were used. Mice obtained from the Animal House, AIIMS, New Delhi, and housed in a pathogen-free specialized small animal facility with a 12 h dark–light cycle. All the animals were treated with utmost human care and had free access to food and water. Mice were euthanized by cervical dislocation, a method authorized by InstitutionalAnimal Ethical Committee (file no- 100/IAEC-1/2018), AIIMS, New Delhi for sacrificing experimental animals, and observed until all muscle activity and breathing has ceased for at least 120 s. No mice died before meeting the endpoints described.

### Isolation and characterization of macrophages from mice

Macrophages were harvested from Swiss albino mice as previously described [24]. Briefly, mice were killed by cervical dislocation and macrophages were harvested by peritoneal lavage as peritoneal exudate cells (PECs) using chilled serum free culture medium (RPMI 1640). PECs were harvested by adherent purification in plastic petri dishes (Tarson, India) at room temperature for 2 h. Adherent cells were collected and seeded in a flat-bottom culture flask (Tarson, India) at a cell density of 1 × 10^6^ in the culture medium with or without LPS and BD-2 and incubated for time periods of 24 h in a CO_2_ incubator for treatment.

### Flow cytometry assay

Macrophages were harvested from Swiss albino mice as described above [24] and at a cell density of 1 × 10^6^ in the culture medium with or without LPS and BD-2 for 24 h in RPMI 1640 containing 10% FBS at 37 °C in 5% CO_2_ in humidified CO_2_ incubator. Macrophages were suspended in RPMI-1640 with 10% FBS, 0.1% NaN3 and incubated with anti-mouse CD14, an antibody conjugated with PE. Isotype is used as a control conjugated with FITC as per manufacturer's instructions. After washing, cells were suspended in 0.1% PBS containing 0.1% NaN3 and then analyzed with a flow cytometry (BD Biosciences, Mountain View, CA, USA)**.**

### ICC

Macrophages were harvested from Swiss albino mice as previously described [25]. The macrophages were characterized by their cell surface marker CD14. After 24 h, the harvested cells were spread over the pre-coated air-dried poly-lysine coated glass slide, and the slide with cells was kept at room temperature to air dry and fix with 4% PFA for 2 h. The slide was washed 3 times with PBS and incubated with 0.1% Triton X-100 for 15 min, followed by washing with PBS thrice followed by staining with DAPI for 5 min and cells were mounted in anti-fed dye Dabco mounting media. Cell morphology was photographed under a fluorescent microscope and Olympus BX51 microscope in grayscale at 100X magnification.

### Peptide cellular uptake assay

To visualize the localization of BD 2in macrophage cells, an ICC assay was done [25]. For this assay, peritoneal macrophage cells were isolated from Swiss albino mice. A pre-sterilized cover slip was placed in the culture plate and macrophage cells were cultured on it. Then cells were treated with BD 2with specified concentrations. After 24 h of incubation, the cover slip that has adhered macrophage cells were isolated from the culture dish and washed with (1X PBS) and treated with 4% paraformaldehyde for 2 h. After three consecutive wash with (1X PBS) cells containing macrophage cells were air-dried for 30 min and treated with 0.1% Triton X for 10 min. After three wash with (1X PBS) cells were treated with BD 2 antibody and incubated overnight. After three wash with (1X PBS) treated with specific secondary antibody and localization of BD 2 was detected under microscopy.

### MTT assay for dose optimization

MTT assay was performed to determine relative cell viability at 24 and 48 h after treatment of BD-2. Macrophage cells were seeded in 96-well plates at 1 × 10^5^ cells/well for 24 h. After that, cells were treated with BD-2 (10–100 ng) and incubated for 24 and 48 h. The cells were washed with 1X PBS and treated with MTT dye (5 mg/ml) for 3 h. After aspirating MTT dye from the plates, cells were treated with 100 µl/well DMSO and optical density (OD) at 570 nm was taken with a multi-plate reader within 15 min and required concentration for a 50% inhibition of viability (IC50) was calculated. Each experiment was performed in triplicates [25].

### ROS measurement

For this assay, macrophages (20000cells/well) were seeded in a 96-well plate and incubated for 24 h. A solution of 20 µM DCFHDA in 100 µl serum-free media was added in each well and incubated for 1 h. After the removal of the DCFHDA solution, each well was washed twice with serum-free media. However, for the negative control, a pre-treatment of 5 mM NAC was done to block the ROS in the specified wells and later treated with BD 2 and H_2_O_2_ (2 mM). Finally, the fluorescence at excitation/emission (485/538 nm) was detected by multiple readers at 1 and 2 h time points [24].

### Nitric oxide (NO) measurement

The level of NO produced was quantified by determining the accumulation of nitrite using the Griess reagent. The optical density of the samples was measured at 540 nm with an ELISA reader. Briefly, cell supernatant was centrifuged to remove debris and clear supernatant was mixed with an equal volume of Griess reagent. The mixture was incubated in the dark for 40 min. The absorbance was measured at 540 nm using a microplate reader. The concentration of nitrite was determined from the sodium nitrite standard curve [24].

### H_2_O_2_ measurement

The production of H_2_O_2_ was quantified by TISO_4_. Add 50 µl of titanium sulfate reagent per 100 µl of sample. If the sample contains peroxide, it will react with the titanium ions in solution to form pertitanic acid, giving the solution a yellow color. The mixture with the reagent should be stable for several hours. Measure the absorbance at 407 nm using the ELISA plate reader [24].

### Anti-tumor assay

AnnexinV-FITC/PI and CFSE labeledc127ibreast cancer cells were co-cultured with macrophages in a transwell chamber with the medium containing 100 ng/ml BD-2. Subsequently, flow cytometry was employed to reveal the percentage of apoptosis, cell division and cell proliferation.

Carboxy-fluorescein succinimidyl ester (CFSE) is cell permeable and covalently couples via its succinimidyl group, to intracellular molecules, -Lysine residues and other amine sources. Due to this covalent coupling reaction, fluorescent CFSE can be retained within cells for extremely long periods. Also, due to this stable linkage, once incorporated within cells, the dye is not transferred to adjacent cells. 50,000 c127i cells were incubated in 300ul of 5 µM CFSE-FITC working solution in PBS for 15 min at 37 °C, protected from light. Excess CFSE was quenched by adding 2 ml complete DMEM culture media containing 10% FBS and incubating for 10 min at room temperature. The cell pellets were resuspended in pre-warmed PBS and washed twice with PBS and centrifugation at 1200 rpm for 5 min at room temperature. C127i cells labeled with CFSE-FITC were resuspended in pre-warmed complete DMEM culture media and seeded in culture dish.

### Gene expression analysis

Total RNA was extracted from the MΦ harvested from control and experimental mice with RNAiso Plus reagent as instructed by the manufacturer (Takara, Japan). High-quality RNA (as estimated by absorbance ratio A260/280P1.8) from different groups were resolved on 1% agarose gel and stained with ethidium bromide to check the integrity of 18S and 28S rRNA using a UV transilluminator.

The total RNAs were used for cDNA synthesis using a kit (BIO-RAD, US), where total RNA (1 µg/20 µl reaction) was implied and further processes were followed as directed by the manufacturer. For real-time PCR, iTaq Universal SYBR Green Supermix was used (BIO-RAD, US). Reaction mix preparation and thermal cycling protocol was followed as directed by manufacturer guidelines. The primers used for the reaction are listed in Tables 1 and 2.

**Table 1:** Cytokine sequences used in this study

Genes	Sequences		Tm (°C)
IFN- gamma	Forward	TTC TTC AGC AAC AGC AAG GC	56.2
	Reverse	ACT CCT TTT CCG CTT CCT GA	56.2
IL-1α	Forward	CCG TGT TGC TGA AGG AGT TG	56.4
	Reverse	GTG CAC CCG ACT TTG TTC TT	55.9
IL- 6	Forward	ACA AAG CCA GAG TCC TTC AGA	56.0
	Reverse	TGG TCC TTA GCC ACT CCT TC	56.3
CD 32	Forward	GAC ACA GCA CCA GTC CAA GA	57.2
	Reverse	CAG TTT TGG CAG CTT CTT CC	54.5
TNF-α	Forward	ACC CTC ACA CTC ACA AAC CA	56.4
	Reverse	GGC AGA GAG GAG GTT GAC TT	56.6
CD 204	Forward	GCC CCA GAG CAA AAA TAT GA	53.1
	Reverse	TGG CTC AAG CTG TTG TCA TC	55.5
IL-3	Forward	GCCTGCCTACATCTGCGAAT	54
	Reverse	GGTTAGGAGAGACGGAGCCA	56
IL-10	Forward	GCTGCCTGCTCTTACTGACT	54
	Reverse	GGGGCATCACTTCTACCAGG	56
TGF-β	Forward	GCTGAGCACCTTTTTGCTCC	54
	Reverse	GATGGCATTTTCGGAGGGGA	54
iNOS	Forward	CTATGGCCGCTTTGATGTGC	59.97
	Reverse	CAACCTTGGTGTTGAAGGCG	59.97
GAPDH	Forward	CGT CCC GTA GAC AAA ATG GT	54.9
	Reverse	TTG ATG GCA ACA ATC TCC AC	53.3

**Table 2:** Chemokine sequences used in this study

Genes	Sequences		Tm (°C)
CXCL-1	Forward	GGTGAGGACATGTGTGGGAG	56
	Reverse	CGAGACCAGGAGAAACAGGG	56
CXCL-5	Forward	CTGCTGCTTTGCCTACCTCT	54
	Reverse	TCTTCTCTGGGTTGGCACAC	54
CXCL-15	Forward	TTGGAGCCAAGGCAAGAACA	52
	Reverse	AATGGAGAGGCATCCGGTTC	54
CCL-24	Forward	GGTCCCTGTCATGCTTCTGG	56
	Reverse	GAGTAGCAGCAGGTGAGTGG	56
CCL-5	Forward	TGCTCCAATCTTGCAGTCGT	52
	Reverse	GCAAGCAATGACAGGGAAGC	54
GAPDH	Forward	CGT CCC GTA GAC AAA ATG GT	54.9
	Reverse	TTG ATG GCA ACA ATC TCC AC	53.3

### Statistical analysis

Data were expressed as the mean ± SE. *P* value < 0.05 was considered significant. All graphs were generated using the Graph Pad Prism software. The multigroup comparisons of means were carried out by one-way analysis of variance (ANOVA) test. Student t-test was used for comparison between groups.

## Data Availability

All data generated or analyzed during the current study available from the corresponding author on reasonable request.

## Abbreviations

TAMs: Tumor associated
TNF: Tumor necrosis factor
PECs: Peritoneal exudate cells
NO: Nitric oxide
PECs: Peritoneal exudate cells
ROS: Reaction oxygen species
CFSE: Carboxy-fluorescein succinimidyl ester
